# The 2024 WHO bacterial priority pathogens list: a critical evolution from a global One Health perspective

**DOI:** 10.1016/j.soh.2025.100145

**Published:** 2025-12-29

**Authors:** Subhankar Mukhopadhyay, Ye Peng, Hein Min Tun

**Affiliations:** a The Jockey Club School of Public Health and Primary Care, Faculty of Medicine, The Chinese University of Hong Kong, Hong Kong Special Administrative Region of China; b System Microbiology and Antimicrobial Resistance (SMART) Lab, Li Ka Shing Institute of Health Sciences, Faculty of Medicine, The Chinese University of Hong Kong, Hong Kong Special Administrative Region of China; c Microbiota I-Center (MagIC), Hong Kong Special Administrative Region of China

**Keywords:** Bacterial priority pathogens list, Antimicrobial resistance, One Health

## Abstract

The 2024 WHO Bacterial Priority Pathogens List (WHO BPPL) is a critical tool for refining global antimicrobial resistance (AMR) strategy, prioritizing 24 bacteria with a focus on Gram-negatives and community threats like *Salmonella* Typhi. This perspective examines its One Health implications. While the 2024 WHO BPPL effectively guides research and development (R&D), policy, and infection control through vaccines and water, sanitation and hygiene (WASH) programs, its human-centric approach underrepresents critical agricultural and environmental AMR drivers. Surveillance biases towards high-income countries and the inherent challenges of cross-sectoral monitoring—given the distinct niches of pathogens like *Enterococcus faecium* and *Shigella*—further limit its scope. We call for integrating zoonotic and environmental metrics, strengthening global surveillance (e.g., Global Antimicrobial Resistance and Use Surveillance System [GLASS]), and accelerating development of novel therapies to advance a more equitable and holistic AMR response.

## 1 Introduction

The World Health Organization's (WHO) Bacterial Priority Pathogens List (BPPL) has been a cornerstone in the global efforts to combat antimicrobial resistance (AMR) since its inception in 2017 [1]. The latest iteration, detailed in the study by Sati et al. [2] and published in *The Lancet Infectious Diseases*, represents a timely and evidence-driven update that refines prioritization of antibiotic-resistant bacteria to steer research, development, and public health interventions. Using a multi-criteria decision analysis (MCDA) framework, the authors evaluated 24 resistant pathogens across eight criteria, incorporating fresh data from systematic reviews, global burden estimates, and expert surveys. This results in a tiered list (critical, high, and medium) that underscores the enduring dominance of Gram-negative bacteria and rifampicin-resistant *Mycobacterium tuberculosis* while highlighting emerging threats in community settings [2]. Far beyond a mere refresh, this 2024 WHO BPPL offers a blueprint for targeted action in an era where AMR claims millions of lives annually.

The study's methodology builds robustly on its predecessor [1], addressing previous limitations such as regional data biases and the integration of non-fatal burden metrics like years lived with disability (YLD) [2]. By weighting criteria through a preferences survey of 78 international experts via the Potentially All Pairwise Rankings of all possible Alternatives (PAPRIKA) method, the authors ensure a balanced, consensus-driven scoring system. High inter-rater agreement (Spearman's rank correlation and Kendall's coefficient both at 0.9) and sensitivity analyses affirm the rankings' stability, providing testaments to the robustness of the final list like carbapenem-resistant *Klebsiella pneumoniae* (84 % score) at the pinnacle and penicillin-resistant group B streptococci (28 %) at the bottom [2].

## 2 Strengths and advancements in prioritization

The data-centric evolution of this upgrade is among its most admirable features. At least 13 new antibiotics have been licensed for priority infections since 2017 [2], marking a modest but noticeable change in the AMR setting. Incorporating 10-year resistance trends and an assessment of the antibiotic pipeline, the 2024 list underscores a sobering reality. It confirms that carbapenem-resistant Gram-negative bacteria, notably *K. pneumoniae* and *Acinetobacter baumannii*, persist in the critical tier, due to their high mortality rates, transmissibility, and a dire lack of effective treatments. The inclusion of rifampicin-resistant *M. tuberculosis* in this tier is particularly poignant, reflecting the intersection of AMR with tuberculosis control efforts in low- and middle-income countries (LMICs), where diagnostic and therapeutic gaps exacerbate the burden [3].

Notably, the list elevates community-acquired pathogens, such as fluoroquinolone-resistant *Salmonella enterica* serotype Typhi (72 %) and *Shigella* spp. (70 %), to the high tier [2]. This shift acknowledges the growing threat of AMR in common infections, often linked to inadequate water, sanitation, and hygiene (WASH) infrastructure [4]. Carbapenem-resistant *Pseudomonas aeruginosa*'s shift from critical to high reflects regional data adaptability and underscores the value of granular surveillance. Such nuances prevent overgeneralization and allow for tailored regional strategies, a marked improvement over the 2017 version [1].

The inclusion of preventability and transmissibility criteria rightly promotes upstream solutions like vaccines, infection prevention, and control steps. The list's rankings help target responses, from hospital stewardship for high-priority vancomycin-resistant *Enterococcus faecium* to the targeted monitoring for medium-tier pathogens (e.g., macrolide-resistant group A streptococci).

## 3 Alternative perspectives: beyond bacteria and towards holistic strategies

A potential limitation of the 2024 WHO BPPL is its focus on human health, which overlooks the “One Health” drivers of AMR [5]. For instance, the rise of carbapenem-resistant Enterobacterales is often linked to agriculture and environmental contamination—dimensions not captured by the current criteria. Furthermore, environmental reservoirs of AMR genes in soil, water, and wildlife microbiomes pose underappreciated risks by acting as amplifiers for human and animal exposure. Despite calls for longstanding monitoring since 2016 [6], these sectors remain unbalanced in One Health frameworks, neglecting pathogens such as environmental *Pseudomonas* strains. This gap underscores the necessity for the Quadripartite's One Health Priority Research Agenda for AMR (AMR-OHPRA), which promotes cross-sectoral integration by prioritizing transmission dynamics at human–animal–environment interfaces and optimized monitoring [5]. Incorporating metrics on zoonotic risk and environmental spread, informed by robust metagenomic surveillance would strengthen future lists [5].

Despite the BPPL's guidance, pipeline progress remains slow [2], highlighting the critical need for pull incentives such as subscription-based reimbursements, to ensure market returns for developers and to stimulate research and development (R&D). Optimism lies in diversifying beyond traditional antibiotics; novel modalities like bacteriophages and monoclonal antibodies offer promising avenues for treating top-tier superbugs. Furthermore, the list's implicit support for vaccines could be formalized by explicitly prioritizing vaccine development, leveraging the proven model of typhoid conjugate vaccine successes [7].

Data limitations, acknowledged by the authors, present another avenue for discourse. Reliance on surveillance from high-income settings may undervalue AMR burdens in LMICs, where underreporting skews incidence and resistance trends. Enhancing global networks like Global Antimicrobial Resistance and Use Surveillance System (GLASS) [8] could address this, ensuring the next BPPL captures a more equitable worldview. Similarly, the genetic expansion of leading carbapenem-resistant *K*. *pneumoniae*, driven by high-risk clones like ST11-KL64 and plasmid-mediated resistance genes, warrants further attention [9]. Neglecting subspecies like *Klebsiella quasipneumoniae*, often misidentified as *K. pneumoniae*, risks underestimating their clinical impact and distinct resistance profiles [10]. Accurate identification via genomics or rapid identification systems (e.g., MALDI-TOF) is crucial to refine surveillance and treatment [10]. Extending One Health surveillance is vital yet challenging, as pathogens like hospital-centric *E. faecium* and environmentally persistent *Shigella* occupy distinct niches across sectors.

## 4 Implications and call to action

The 2024 WHO BPPL is a positive stride, providing an authoritative, transparent, and globally relevant roadmap for addressing AMR. Its implications extend to policy, with governments and funders urged to align investment for instance, prioritizing carbapenem-resistant Gram-negatives in R&D grants or bolstering WASH for community pathogens. For clinicians, it reinforces stewardship, emphasizing reserve antibiotics for critical cases. Ultimately, the list affirms that AMR is a tractable problem, with the BPPL itself catalyzing progress since 2017.

Successful outcomes, however, require rigorous implementation. Stakeholders must translate priorities into tangible outcomes by accelerating the antibacterial pipeline, bridging data gaps, integrating zoonotic and environmental metrics into future criteria, enhancing GLASS with LMICs and microbiome data, and prioritizing vaccine R&D per WHO guidelines. Future strategies must also consider emerging threats like climate change impacts, which could exacerbate pathogen spread through altered ecosystems. In endorsing this update, we affirm that prioritizing pathogens is not just scientific exercise but a moral imperative to safeguard global health equity.

## CRediT authorship contribution statement

**Subhankar Mukhopadhyay:** Writing – original draft, Formal analysis, Conceptualization. **Ye Peng:** Writing – review & editing, Formal analysis. **Hein Min Tun:** Writing – review & editing, Supervision, Resources, Funding acquisition, Formal analysis, Conceptualization.

## Funding

HMT acknowledges funding from the Fleming Fund Fellowship Scheme Phase 2 (No: TM2419543), HMRF Fellowship Scheme (No. 04180077) and the Theme-based Research Scheme grant from the Research Grants Council of the Hong Kong Special Administrative Region, China (No. T21-705/20-N).

## Declaration of competing interest

The authors declare the following financial interests/personal relationships which may be considered as potential competing interests: Hein Min Tun reports financial support was provided by Fleming Fund Fellowship Scheme Phase 2. Hein Min Tun reports financial support was provided by HMRF Fellowship Scheme, and the Theme-based Research Scheme grant from the Research Grants Council of the Hong Kong Special Administrative Region, China. If there are other authors, they declare that they have no known competing financial interests or personal relationships that could have appeared to influence the work reported in this paper.
